# A System of Human Anatomy, General and Special

**Published:** 1848-10

**Authors:** 


*A System of Human Anatomy, General and Special.* By ERASMUS WILSON, M. D. *Fourth American from the last London edition.* Edited by PAUL B. GODDARD, A. M., M. D., Professor of Anatomy and Histology in the Franklin Medical College of Philadelphia. 8vo. pp. 576. Lea & Blanchard. Philadelphia: 1848.

In this excellent work, all the improvements which have been made public since the appearance of the former edition, are carefully noted, and many additional wood-cuts added. The present edition contains 251 illustrations by Gilbert, and is every way deserving of approbation.

